# Supplementary material for: Machine learning approaches to enhance diagnosis and staging of patients with MASLD using routinely available clinical information
Source: PLoS One. 2024 Feb 29;19(2):e0299487. doi: 10.1371/journal.pone.0299487 (PMC10903803; doi:10.1371/journal.pone.0299487)
Supplement: S1 File — (DOCX) [file pone.0299487.s001.docx]

**Supplementary Material**


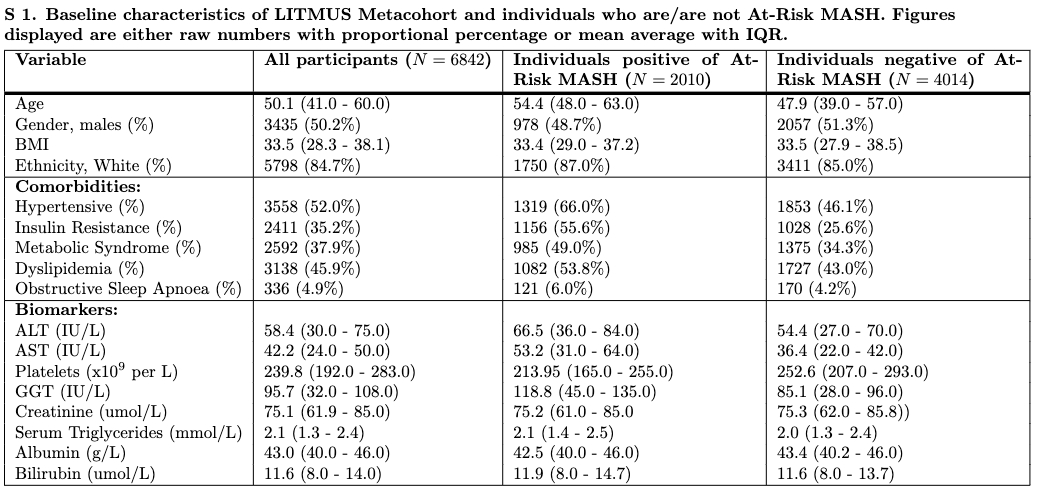
Summary statistics of the LITMUS Metacohort and individuals who are positive and negative of At-Risk MASH at baseline assessment:

Note: The total number of positive and negative At-Risk MASH (and indeed for all other target variables) cases does not add up to the total in the entire LITMUS Metacohort, this is due to individuals sometimes not having certain information that is necessary to derive target variables recorded.
